# Mobilising communities to address alcohol harm: an Alcohol Health Champion approach

**DOI:** 10.1177/1757913919899700

**Published:** 2020-03-23

**Authors:** Cathy Ure, Liz Burns, Suzy C Hargreaves, Margaret Coffey, Suzanne Audrey, Kiran Kenth, Kate Ardern, Penny A Cook

**Affiliations:** Research Project Manager – Public Health, School of Health and Society, University of Salford, Allerton Building, Frederick Road, Salford M6 6PU, Manchester, UK; Lecturer in Mental Health Nursing, School of Health and Society, University of Salford, Salford, UK; Research Assistant, Public Health, School of Health and Society, University of Salford, Salford, UK; Reader in Public Health, School of Health and Society, University of Salford, Salford, UK; Senior Research Fellow in Public Health, Population Health Sciences, Bristol Medical School, University of Bristol, Bristol, UK; Director of National and Regional Programmes, The Royal Society for Public Health, London, UK; Director of Public Health, Wigan Council and Greater Manchester DPH for Drugs and Alcohol Harm Reduction; Professor in Public Health, School of Health and Society, University of Salford, Salford, UK

*In this article, Cathy Ure et al. look at engaging communities in order to reduce alcohol harms. By training Alcohol Health Champions, individuals can support vulnerable friends and family, and work within their communities to influence policy and promote change.*

## Background

Globally, harmful drinking results in six deaths every minute.^1^ The evidence indicates that restricting the availability of alcohol, and early identification and brief advice (IBA) are effective interventions to reduce alcohol harm.^2^ Furthermore, recent work suggests that there is a need to engage communities in actions to reduce alcohol harm.^3^ In order to tackle the high social and economic cost of alcohol, estimated to be £1.3 bn per year or £500 per resident,^4^ the UK city region of Greater Manchester (GM) implemented an innovative programme to reduce alcohol harm in September 2017. This asset- and place- based community development approach^6^ – called *Communities in Charge of Alcohol* (CICA) – aims to reduce alcohol harm in specific deprived areas across 10 local authorities.^7^

**Figure:**
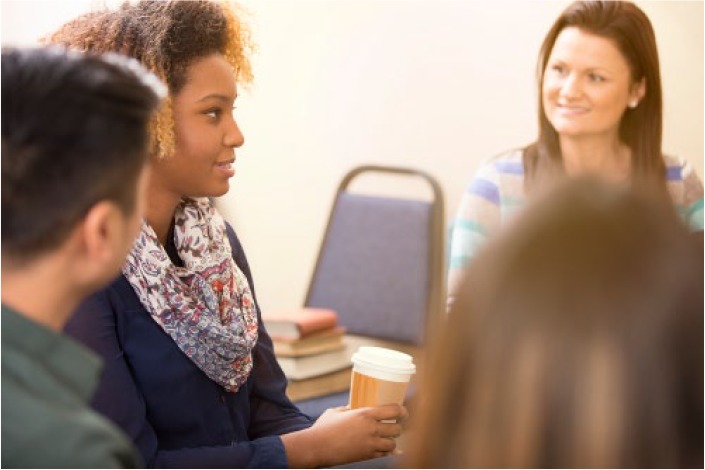


The CICA programme trains local volunteers within specific communities to become alcohol health champions (AHCs) – the first time to our knowledge that such a role has been established. This article introduces the role of AHCs, talks about who they are, and provides a glimpse into their experiences to date.

**Table:** 

**Alcohol Health Champions are trained to:**	
• Have informal conversations about alcohol and health with family, friends, and colleagues and to use the Audit-C (an alcohol harm assessment tool in the form of a scratch card with three questions around alcohol consumption); • Support people to reduce drinking through brief advice or guiding them towards specialist services; • Provide local support for communities to get involved with licensing decisions by helping them raise issues with the local authority about venues selling alcohol; • Work with other members of the community and professionals to influence alcohol policy/availability in their community; • Train others to become AHCs (first generation AHCs only).	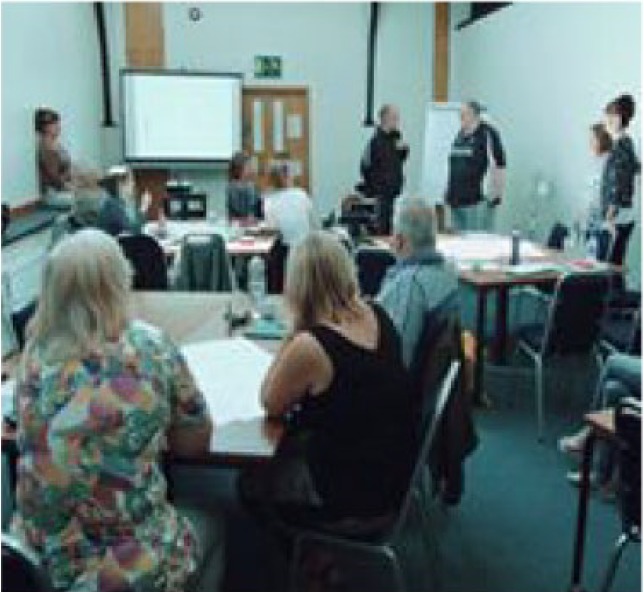

## The Role of AHCs

AHCs are lay people living or working in the areas where CICA was implemented and who have gained the RSPH Level 2 Award ‘Understanding Alcohol Misuse’ accreditation as a result of partaking in the CICA training programme. This award – a bespoke design for CICA – entails two days of learning in relation to alcohol awareness and giving brief advice. Unique to the AHC role is also learning about the Licensing Act 2003. This knowledge enables community members to build relationships with local licensing officers, have a voice, and influence licensing decisions locally. The initial cohort of AHCs also received Train the Trainer input to enable them to train future volunteers to become AHCs. AHCs are recruited and supported by a locally assigned CICA co-ordinator (staff already employed in lifestyle provider services or Tier 3 alcohol services).

## What Has Happened So Far?

In the first 18 months, 123 new health volunteers were trained as AHCs. Motivations for becoming an AHC included being a ‘concerned relative’, wanting to help others, personal experience of alcohol dependence, a general desire to learn more about alcohol, working in the local community, and/or gaining a qualification. The AHCs’ predominant focus to date has been on providing brief advice within their communities. Data from five areas show that: 65 community events were attended by AHCs; 1129 conversations took place with members of their communities; and 249 AUDIT-C assessments completed.^8^ Experiences of getting involved in licensing were less commonly reported by AHCs, but individual stories highlighted examples where AHCs had reported issues to local licensing leads and had raised awareness of local licensing powers within the community. AHCs cited concerns about being publicly identifiable as a barrier to engagement in formal licensing processes.

## What Benefits Do AHCs Report?

It was evident from early in the programme that there was considerable social value gained from becoming an AHC. Inspiring stories relating to the personal benefits to AHCs include: gaining permanent employment; increased confidence; developing positive, supportive friendships; widening social networks; reduced personal levels of alcohol use; and feeling good about making a difference. Indeed, one of the challenges experienced by local CICA co-ordinators has been retaining AHCs as they move on to utilise their new-found skills elsewhere, in training or employment.

**Figure:**
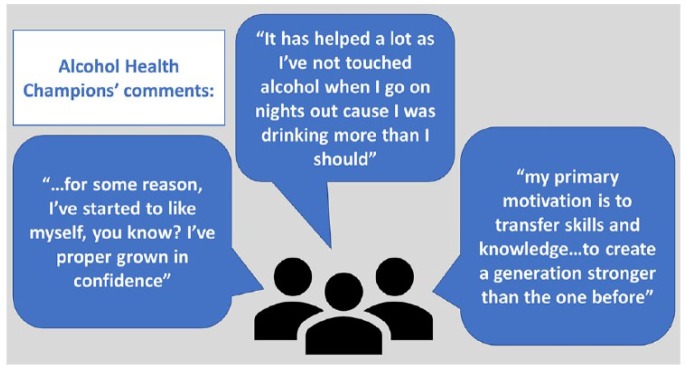


## Initial Reflections

We are in the process of evaluating the impact CICA has on reducing alcohol harm within the communities where it was rolled out.^7^ We have learnt that CICA is a complex intervention to launch and embed into small communities, and have identified key barriers and facilitators which have affected the implementation, recruitment, training, and ongoing support of AHCs. It has become evident that the effective implementation of the AHC training, and integration of the role to deliver alcohol harm reduction activities into local plans is a process which needs time to bed in, facilitated by ongoing support from local commissioners. It is really pleasing that, two years after initial launch, five local authorities across GM continue to recruit and train AHCs. Locally, the value of AHCs is recognised with local co-ordinators inspired by their AHCs’ desire to tackle alcohol harm and the personal benefits to health and wellbeing gained by the AHCs themselves. More information about the role of AHCs is available at http://hub.salford.ac.uk/communities-in-charge-of-alcohol/alcohol-health-champions/. The CICA protocol is available at https://bmcpublichealth.biomedcentral.com/articles/10.1186/s12889-018-5410-0.
